# Assessing Tissue Transmission of Hepatitis C Virus From Viremic Donor to Seronegative Kidney Transplant Recipients: A Case Series

**DOI:** 10.3389/ti.2023.11110

**Published:** 2023-07-05

**Authors:** Antonio Franco, Carla Gosalvez, Adelina Gimeno, Migul Trigueros, Noelia Balibrea, Francisco Javier Perez Contreras

**Affiliations:** ^1^ Department of Nephrology, Hospital General Universitario Dr. Balmis, Alicante, Spain; ^2^ Department of Microbiology, Hospital General Universitario Dr Balmis, Alicante, Spain; ^3^ Department of Pathology, Hospital General Universitario Dr Balmis, Alicante, Spain

**Keywords:** kidney transplantation, hepatitis C virus, viremic donor, transmission, seronegative recipient

## Abstract

The transmission of hepatitis C virus from viremic donors to seronegative recipients of kidney transplantation is well documented. Pre-transplant administration of direct-acting antivirals prevents viremia, but the seroconversion rate is high. We studied the transmission of the virus through the transplanted tissue by determining viral RNA in 15 kidneys from 8 deceased viremic donors, 5 males and 3 females aged 52.3 ± 15 years. HIV positive donors and active intravenous drugs abusers were discarded to avoid possible window periods in the virus transmission. Recipients, 9 males and 6 females aged 52.7 ± 18 years, were treated with glecaprevir/pibrentasvir for 8 weeks and received immunosuppression with thymoglobulin, tacrolimus, sirolimus and prednisone. Hepatitis C Virus was detected in 9 of the 15 histological samples analyzed but viremia was detected in no recipient at day 1 and 7 post–transplantation and 12 weeks after the treatment. However, 13 of the 15 recipients had seroconverted within 1 month. In conclusion, Hepatitis C virus was detected in a significant proportion of tissue of kidney grafts from viremic donors, but treatment with direct-acting antivirals avoids the transmission of the virus from donor to recipient. Then Donor pools should be expanded.

## Graphical Abstract

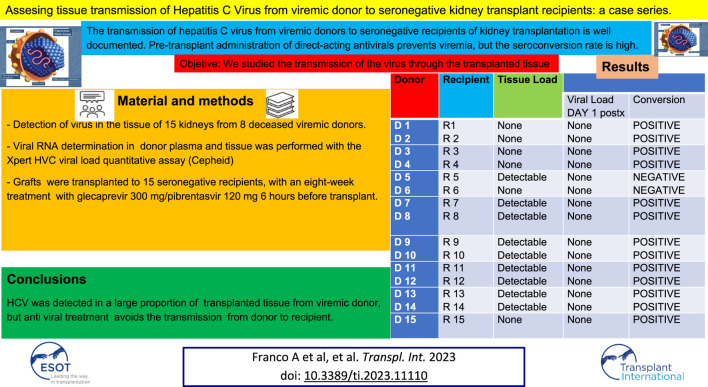

## Introduction

Transmission of hepatitis C virus (HCV) from viremic donor to seronegative recipient via kidney transplantation is well documented [1, 2]. Administration of direct-acting antivirals (DAA) to the recipient pre-transplant prevents post-transplant viremia and possibly transmission of infection [3, 4]; however, starting treatment in the post-transplant period prevents neither viremia nor the consequent transmission [5, 6]. In addition to detecting viral particles in different extrahepatic compartments, including kidney tissue, several studies have also observed viral replication [7–13], which could enable the transmission of the infection in the absence of viremia through the tissue of kidney grafts.

The aim of this study was to assess HCV transmission through the tissue of kidney grafts from viremic donors to seronegative recipients treated with DDA.

## Materials and Methods

### Study Design and Population

This study is a case series on adult kidney transplant recipients from HCV viremic donors undergoing transplantation from March 2018 and December 2019 in the Hospital General of Alicante (Spain).

We determined the presence of HCV in the tissue of 15 kidneys from 8 deceased viremic donors (one graft was not included in the study because it was transplanted to a seropositive recipient). HIV positive donors and active intravenous drugs abusers were discarded to avoid possible window periods in the virus transmission.

### Procedures

The grafts were transplanted to 15 seronegative recipients, who started an eight-week course of treatment after breakfast with glecaprevir 300 mg/pibrentasvir 120 mg 6 h prior to transplantation.

Plasma viral load was determined in donors within a few hours prior to transplantation, and in recipients at day 1 and 7 post–transplantation and 12 weeks after the treatment. Antibodies against HCV were measured in the serum of transplant patients 1 month after transplant.

Tissue samples were extracted in the operating room for diagnostic purposes using an 18G biopsy needle (Biopince full core biopsy instrument). Tissue cylinders were 18 mm–22 mm long and 1 mm thick. They were paraffinized for pre-transplantation histological study and then deparaffinized for virus detection. The deparaffinized tissue was washed three times with sterile saline and homogenized with 2 mL of sterile distilled water.

Viral RNA determination in donor plasma and tissue was performed with the Xpert HVC viral load quantitative assay (Cepheid), which has a quantification range of 10 IU/mL to 4–6 IU/mL. The Xpert HCV Viral Load Assay is a polymerase chain amplification (PCR)-based assay with no need for batch processing of samples and with a result available in 105 min. This test was used in donor samples because it allows quick results on demand. In biopsy samples it was used due to its higher sensitivity. This method employs a reverse transcription polymerase chain reaction technique (RT-PCR) that uses fluorescence to detect and quantify the RNA of HCV genotypes 1 to 6.

In recipients, HCV viral load was determined using RT-PCR with the HCV COBAS AmpliPrep/COBAS TaqMan quantitative technique, v2.0, whose lower detection limit is 15 IU/mL. This test is designed for batch testing of multiple specimens within a run. Antibodies against HCV were measured in the serum of transplant patients using the Roche Diagnostics Elecsys Anti-HCV II assay.

### Ethic Issues

The present study was performed n accordance with the Declaration of Helsinki and was consistent with the Principles of the Declaration of Istambul on Organ Trafficking and Transplant Tourism. This study received approval from the Hospital of Alicante Review Board (record 2021-04, 28 April 2021).

The strategy of kidney transplant from viremic and non-viremic hepatitis C positive donors into negative recipients was conducted under The Spanish consensus document coordinated by the National Transplant Organization (ONT) [14]. All recipients had signed an informed consent when were enroled in the waiting list.

### Statistical Analysis

This is a descriptive analysis. Categorical data are shown as absolute numbers and their frequencies, and metrics used for continuous data with relative dispersión.

## Results

Table 1 shows donor and recipient demographics and post-transplant outcomes at 3 years.

**TABLE 1:** Donor and recipient characteristics and outcome at 3 years.

	Donor	Recipient
Age (years)	52.3 ± 15	52.7 ± 18
Male gender	5/8: 62.5%	9/15: 60%
Virus Genotipe 1a	4	na
1b	3	
3	1	
Blood Group A	2	4
B	1	2
AB	0	0
0	5	9
End stage cause	na	
glomerular disease		2
interstitial nephritis		4
Lupus		2
polycystic disease		3
Hypertension		1
diabetes nephropathy		3
Graft. Survival at 3 years	na	93.3%
Patient survival at 3 years	na	93.3%

Na, No applicable.

All recipients received immunosuppression with thymoglobulin, tacrolimus, sirolimus and prednisone.

Table 2 shows the plasma viral load in the donors pre-transplant and in the recipients on day 1 and 7 post-transplant and 12 weeks after treatment, the serological results against HCV at 1 month, and the detection of HCV in the transplanted tissue. Plasma viral load in all donors was significant, and in recipients it was undetectable on days 1 and 7 post-transplantation and 12 weeks after treatment. However, 13 of the 15 recipients had seroconverted within 1 month. HCV was detected in 9 of the 15 histological samples analyzed. In all cases where viral RNA was detected, the concentration was less than 10 IU per tissue sample.

**TABLE 2:** Plasma viral load in donors pre-transplant and in recipients on day 1 and 7 post-transplant and 12 weeks after treatment, serological results against virus at 1 month, and detection of virus in the transplanted tissue.

Donor	Recipient	Donor plasma viral Load (UI/mL)	Recipient plasma viral load (UI/mL)			Kidney viral load	Seroconversion
			Day 1	Day 7	12 weeks post transplant		
D 1	R1	4,000	UNDETECTABLE	UNDETECTABLE	UNDETECTABLE	UNDETECTABLE	POSITIVE
D 2	R 2		UNDETECTABLE	UNDETECTABLE	UNDETECTABLE	UNDETECTABLE	POSITIVE
D 3	R 3	470,000	UNDETECTABLE	UNDETECTABLE	UNDETECTABLE	UNDETECTABLE	POSITIVE
D 4	R 4		UNDETECTABLE	UNDETECTABLE	UNDETECTABLE	UNDETECTABLE	POSITIVE
D 5	R 5	1,400,000	UNDETECTABLE	UNDETECTABLE	UNDETECTABLE	DETECTABLE	NEGATIVE
D 6	R 6		UNDETECTABLE	UNDETECTABLE	UNDETECTABLE	UNDETECTABLE	NEGATIVE
D 7	R 7	1,300,000	UNDETECTABLE	UNDETECTABLE	UNDETECTABLE	DETECTABLE	POSITIVE
D 8	R 8		UNDETECTABLE	UNDETECTABLE	UNDETECTABLE	DETECTABLE	POSITIVE
D 9	R 9	180,000	UNDETECTABLE	UNDETECTABLE	UNDETECTABLE	DETECTABLE	POSITIVE
D 10	R 10		UNDETECTABLE	UNDETECTABLE	UNDETECTABLE	DETECTABLE	POSITIVE
D 11	R 11	1,300,000	UNDETECTABLE	UNDETECTABLE	UNDETECTABLE	DETECTABLE	POSITIVE
D 12	R 12		UNDETECTABLE	UNDETECTABLE	UNDETECTABLE	DETECTABLE	POSITIVE
D 13	R 13	4,500,000	UNDETECTABLE	UNDETECTABLE	UNDETECTABLE	DETECTABLE	POSITIVE
D 14	R 14		UNDETECTABLE	UNDETECTABLE	UNDETECTABLE	DETECTABLE	POSITIVE
D 15	R 15	450,000	UNDETECTABLE	UNDETECTABLE	UNDETECTABLE	UNDETECTABLE	POSITIVE
D16	R 16						

All recipients completed treatment with DAA without reported adverse events or treatment interruptions. We do not modify DAA posology during treatment. The interactions of DAA with Tacrolimus and sirolimus were managed with the monitorization of their plasmatic levels.

## Discussion

In our study, seroconversion occurred in most kidney transplant recipients with viremic donors, despite the absence of viremia in the immediate post-transplant period (Table 2). These results are consistent with previous reports [3, 4] and indicate that seronegative recipients had contact with the virus.

The presence of HCV in the extrahepatic tissue of viremic patients has scarcely been studied, but several authors have described it in gastrointestinal mucosa [7], lymph nodes [8, 9], bone marrow [10], the central nervous system [11], the pancreas, heart, and even kidney [12, 13]. In addition, some authors have observed the presence of negative-polarity RNA in extrahepatic tissue [7, 12, 15], which acts as an intermediary in the replication of the virus, confirming the possibility of transmitting the active infection [15].

The HCV detection rate in different extrahepatic tissues is low, and obviously lower than that detected in the liver [15] according to the authors, these findings suggest that levels of HCV infection and replication at the extrahepatic level are low, but still sufficient to transmit infection [15].

In our study, we detected viral RNA in most of the donor kidney tissue samples by means of a quantitative RT-PCR, Xpert HVC Viral Load (Cepheid). We did not look for evidence of viral replication, which has already been demonstrated by other authors [7, 12, 15]. In all cases where viral RNA was detected, the concentration was under 10 IU/mL.

We chose this technique because it is easy to use and very sensitive, with a detection limit below 10 IU/mL. As Wrobleswska et al. [16] have described, the volume of the samples determines the sensitivity of the technique. The number of viral particles in extrahepatic tissues is very low [13, 16], and in our case, the samples were small.

The technique used is commercially available and validated for serum and plasma. Currently, there is no validated technique for tissue [13].

Yan et al. assessed HCV by *postmortem* RT-PCR of extrahepatic tissue in nine patients with severe viral hepatitis, detecting its presence in the kidney, pancreas, heart, and intestine in all cases, and its replication by negative-polarity RNA in some, including the kidney; authors concluded that HCV can infect and replicate in various extrahepatic tissues [12].

Gelpi et al. [17] reported an experience similar to ours, but with different and perhaps complementary results. These authors ruled out the presence of occult HCV infection by kidney graft biopsy in three seronegative recipients who had not taken DAA when receiving the transplant from seropositive, non-viremic donors. This is a different case from our study, in that our recipients did receive treatment because donors were viremic. Taken together, these studies provide a more complete picture on the silent transmission of HCV through kidney tissue, with no transmission occurring with non-viremic donors and transmission in the case of viremic donors [18].

These data are corroborated by Shike et al. [13], who studied HCV in the kidney tissue of 14 seropositive donors rejected for transplantation. In the 11 donors with a positive plasma load, investigators detected viral RNA by RT-PCR in 16 of the 22 kidneys (72.7%), data similar to ours. However, in the remaining three donors with a negative plasma load, none of the six grafts showed viral RNA, in keeping with Gelpi’s results [17]. Compared to our study, Shike et al.’s [13] has the advantage of having a large amount of tissue on which the virus could be detected, since it included kidneys rejected for transplantation instead of on graft cylinders for transplantation.

In our group, viral RNA was detected in D5 kidney tissue, but not in D6, from the same donor (Table 2), Shike’s experience is similar to ours, as they also detected the virus in the tissue of one but not both kidneys in two of their donors [13]. These authors [13], like us, attribute the cases of non-detection of the virus in tissue to the low viral load present in extrahepatic tissues [16] and to the lower sensitivity of the tissue detection technique [13].

Shike et al. [13] establish a correlation between the amount of viral load in the donor’s plasma and the presence of virus in the tissue, concluding that the higher the viral plasma load, the more likely the virus will be detected in tissue and the larger the amount. Data from our study seem to confirm this conclusion: in the cases where no virus was detected in tissue (D1, D2, D3, D4, D15), the viral plasma load remained under 500,000 IU/mL (Table 2).

The viral concentration in tissue was below the technique’s linear range. However, the detection of viral RNA in most grafts suggests that HCV could be detected in all grafts from viremic donors if the tissue samples were larger, as in the study by Shike [13].

In spite of detecting HVC in plasma and tissue from the donors, the treatment with DAA avoided the transmission of the virus from donor to recipient. As a matter of fact, recipients from viremic donors starting DAA pre-transplant do not present viral replication at any time or only a very low one, but starting DAA after transplant show viral transmission in all the cases. Then, starting DAA pre-transplant should be mandatory [18].

The main strength of our study is the detection of the virus in both, donors through plasma viral load and tissue detection, and in recipients, through plasma viral load and subsequent serology. In contrast, Shike’s study was limited to detection in the donor [13].

The study’s primary limitation is the lack of a validated technique for tissue. The second limitation is the small amount of tissue available for virus detection.

In conclusion, HCV was detected in a large proportion of transplanted tissue from viremic donors, which could facilitate its transmission to recipients, but treatment with DAA avoids the transmission of the virus from donor to recipient. Then Donor pools should be expanded.

## Data Availability

The original contributions presented in the study are included in the article/Supplementary material, further inquiries can be directed to the corresponding author.
